# Sphincter of Oddi and its Dysfunction

**DOI:** 10.4103/1319-3767.37793

**Published:** 2008-01

**Authors:** Prasad Seetharam, Gabriel Rodrigues

**Affiliations:** Department of General Surgery, Kasturba Medical College, Manipal University, Manipal, Karnataka, India

**Keywords:** Bile duct, dysfunction, pancreas, sphincter of Oddi, surgery

## Abstract

Sphincter of Oddi though mostly heard about in ‘anatomy textbooks’ is making its way into surgical practice due to various disease states affecting it and its dysfunction seems to be an important condition to be observed while treating patients with abdominal pain. In this review, we have attempted to discuss all the relevant conditions affecting it, particularly the dysfunction with a detailed literature review.

The sphincter of Oddi (SO) is a structure consisting of smooth muscle fibers that surround the distal common bile duct (CBD), the main pancreatic duct and the ampulla of Vater. Its role is to regulate the flow of bile and pancreatic juices into the duodenum as well as to prevent the reflux of the duodenal contents into the pancreatobiliary system.[1] The abnormalities of SO contractility (sphincter of Oddi dysfunction: SOD) may be related to either the biliary or the pancreatic segments of the sphincter or both.[2] SOD refers to structural or functional disorders involving the biliary sphincter that may result in the impedance of bile and pancreatic juice flow. Up to 20% of patients with continued pain after cholecystectomy and 10–20% of patients with idiopathic recurrent pancreatitis may suffer from SOD. This condition is more prevalent among middle-aged women for unknown reasons.[3]

## ANATOMY

The term SO is named after Oddi who first described the smooth muscle ring found in association with the pancreaticobiliary confluence. However the credit for a detailed anatomic description of the sphincter goes to Boyden.[4] The present concept regarding the musculature of SO is that there is actually a complex of four sphincters composed of circular or spiral smooth muscle fibers surrounding the intramural part of the CBD and pancreatic ducts, and these are the superior sphincter choledochus, inferior sphincter choledochus, sphincter pancreaticus and sphincter of the ampulla.[5]

## PHYSIOLOGY

The musculature of SO is functionally different from the musculature of duodenum. Endoscopic manometric studies have demonstrated that human SO creates a high pressure zone between the bile duct and duodenum.[6] The SO maintains a basal pressure that is considerably higher than the duodenal pressure and approximately 3 mm Hg greater than the pressure in the CBD and pancreas. Further, SO exhibits high-pressure phasic contractions. The amplitude of the phasic contraction is approximately 130 mm Hg and the mean frequency is about 4/min. Most contractions are oriented in an antegrade direction from the CBD towards the duodenum, while a few of them tend to be spontaneous and retrograde. Conflicting reports are available regarding the synchronicity of the high-pressure contractions of SO with phases of migrating motor complex (MMC).[6]

Both neural and hormonal factors influence the SO. During fasting, the SO maintains the high-pressure status to facilitate the filling up of the gall bladder. Further, the activity of SO is coordinated with the emptying of gall bladder that occurs during phase 3 of MMC. This may be a preventive mechanism against the accumulation of biliary crystals during fasting.[7]

The relaxation of SO following meals is predominantly under the influence of Cholecystokinin and secretin. The role of CCK has been further substantiated by studies, which have suggested that cholecystectomy, at least in short term, suppresses the normal inhibitory effect of pharmacological doses of CCK on SO.[8] However, the mechanism of this effect is not known. Besides CCK and secretin, there is evidence to suggest that nonadrenergic and noncholinergic neurons use vasoactive intestinal polypeptide (VIP) as a neurotransmitter to contribute to the relaxation of SO. However, the innervation of the bile duct is not absolutely essential for the functioning of SO as sphincter functioning is well preserved following liver transplantation.[9]

Neurally mediated reflexes link the SO with the gall bladder and stomach to coordinate the flow of bile and pancreatic juice into the duodenum. The cholecysto-SO reflex allows the SO to relax as the gall bladder contracts.[10] Similarly, antral distention causes both gall bladder contraction and SO relaxation.[11]

## PATHOLOGY

A normally functioning SO is very much essential to maintain stability in the hepatobiliary and pancreatic territory. Disturbance in the functioning of SO results in various debatable clinical entities which include (a) SOD, (b) acute pancreatitis and (c) bile duct cysts.

**a) SOD:** SOD is a poorly defined, incompletely understood clinical syndrome. Various terms such as papillary stenosis, sclerosing papillitis, biliary spasms, biliary dyskinesia and postcholecystectomy syndrome have been used synonymously and this further adds confusion to the scenario. To avoid confusion, two types of SOD have been proposed on the basis of pathogenic mechanisms, namely, stenosis and dyskinesia.[12,13]

*SO stenosis:* This is a structural anomaly with narrowing of part or whole of the sphincter as a result of chronic inflammation and fibrosis. This condition may be caused by pancreatitis, injury from gall stone migration through the papilla, trauma from intra operative manipulation of the CBD or non specific inflammatory conditions like adenomyosis.

*SO dyskinesia:* This is an intermittent functional blockage in the high-pressure zone of the sphincter. It results from spasm, hypertrophy or denervation of the sphincter muscle. The condition may reflect a motility disorder of the SO similar to motility disorders elsewhere in the gastrointestinal tract.

**b) Acute pancreatitis:** Manometric studies of SO indicate that motility disorders of SO may be implicated in the aetiopathogenesis of acute pancreatitis.[14]

**c) Bile duct cysts:** It has been hypothesized that bile duct cysts (choledochal cysts) occurring among adults could actually be an acquired condition resulting from SOD.[15] However, the data to support this hypothesis appears to be insufficient.

Among the abovementioned entities that are attributed to a pathological SO, the SOD is the most controversial and debated subject and shall remain the subject of discussion.

**Role of microlithiasis:** Microlithiasis formation occurs mainly in the gallbladder as a result of altered mucosal function and motility. Microlithiasis can also occur in the CBD. Microlithiasis was held responsible for postcholecystectomy pain and acute pancreatitis. However, studies have conclusively proved that microlithiasis can exist in a small percent of individuals in the gall bladder or CBD irrespective of functional status of SO. Thus, microlithiasis appears to have a very small role in the pathogenesis of SOD.[16,17]

## SPHINCTER OF ODDI DYSFUNCTION

### Epidemiology

As the clinical syndrome of SOD is poorly defined and incompletely understood, an accurate estimate of the problem cannot be made. Elevated basal SO pressure has been reported in 40% of patients with gall stones with or without biliary pain.[18] When abnormal liver enzymes were present, 40% of 25 similar patients without ductal stones had an elevated basal SO pressure. In contrast, no basal SO pressure elevation above 30 mm Hg was found in 50 asymptomatic volunteers.[19] Ruffolo and co workers reported that 50% of 81 patients with biliary type pain, intact gall bladders and no evidence of gall stones presented with the delayed emptying of the gall bladder, SOD or both.[20]

Postcholecystectomy pain resembling preoperative biliary pain occurs in 10–20% of patients.[21] SOD has been reported in 9–14% of patients examined for postcholecystectomy pain.[22] When other causes of postcholecystectomy pain have been excluded and SO manometry has been performed in a more carefully screened group, the frequency of SOD is 30–60%.[23] When these patients are classified by Milwaukee Classification [Table 1] for possible SOD, the frequencies of elevated basal SO pressure are 86%, 55% and 28% for patients with types I, II and III suspected SOD, respectively.

**Table 1:** Classic (Milwaukee) and contemporary (modified Milwaukee) classification for presumptive SOD

**Presumptive SOD type**		**Definition**
Biliary type I	Classic	Pain + abnormal hepatic enzymes on 2 occasions + dilated CBD + delayed drainage > 45 min
	Contemporary	Pain + abnormal hepatic enzymes + dilated CBD
Biliary type II	Classic	Pain + 1 or 2 of hepatic enzymes × 2, dilated CBD, delayed drainage ≥ 45 min
	Contemporary	Pain + abnormal hepatic enzyme or dilated CBD
Biliary type III	Classic/contemporary	Biliary type pain alone
Pancreatic type I	Classic	Pain + abnormal pancreatic enzymes on 2 occasions + dilated PD + delayed drainage ≥ 8 min
	Contemporary	Pain + abnormal pancreatic enzymes + dilated PD
Pancreatic type II	Classic	Pain + 1 or 2 of pancreatic enzymes × 2, dilated PD, delayed drainage ≥ 8 min
	Contemporary	Pain + abnormal pancreatic enzyme or dilated PD
Pancreatic type III	Classic/contemporary	Pancreatic type pain alone

### Clinical features

SOD can occur in any age group; however, patients with SOD are typically middle-aged females.[24] It can possibly cause the following clinical conditions:

1. Persistent or recurrent biliary pain in the absence of structural abnormalities following cholecystectomy
2. Idiopathic recurrent pancreatitis
3. Biliary pain in patients with intact gall bladder but without cholelithiasis

The most common presentation of SOD is a disabling abdominal pain, which is experienced in the epigastrium or right upper quadrant. The duration of this pain is variable and may last for 30 min to several hours. This pain can be associated with nausea and vomiting. It can be precipitated by food and narcotics. Jaundice, fever or chills are rarely observed. Physical examination is rarely contributory except for the mild nonspecific abdominal tenderness.

SOD has been described among patients who have undergone liver transplantation,[25] who have acquired immunodeficiency syndrome[26] and hyperlipidemia.[27] The pain of SOD could be of biliary or pancreatic type. The pain commonly accepted to be consistent with “biliary type” SOD is episodic and it lasts for more than 45 min, but not more than several hours and it is perceived in the epigastrium or right upper quadrant, which is analogous to that associated with biliary stone disease.[28] The pain of “pancreatic type” SOD typically is described as postprandial, episodic and prolonged, but not continuous, which is felt in mid to upper abdomen or back.

## INVESTIGATIONS FOR EVALUATION OF THE FUNCTIONING OF SO

The investigations used to assess the functioning of SO fall into three broad categories, namely, (a) pharmacologic tests (b) imaging studies and (c) manometric studies.

### a) Pharmacologic tests

The most widely used pharmacologic test is the morphine-prostigmine provocative test of Nardi.[29] In this test, an intramuscular injection of 10 mg of morphine and 1 mg of prostigmine is given to produce simultaneous spasm of SO and stimulation of exocrine pancreatic secretion. The reproduction of the pain or increase in the pancreatic or liver associated enzymes (amylase/lipase) is considered as a positive test suggestive of SOD. This test also identifies patients who are most likely to benefit from sphincteroplasty and septectomy.

### b) Imaging studies

**1) Ultrasonography:** Following cholecystectomy, a dilated CBD, as detected by sonography, is likely to be a feature of SOD[30] with high predictive value for a favorable outcome after sphincterotomy.[31] The determination of change in the diameter of CBD in response to a fatty meal or cholecystokinin octapeptide may be more useful in the evaluation of SO function.[32] In general, there should be no increase in the diameter of CBD, as measured by ultrasonography. Changes in the sphincter motility would enhance bile flow into the duodenum and decrease the diameter of CBD.[13] A paradoxical increase in CBD diameter reflects abnormal resistance to bile flow and an increase greater than 2 mm is observed among patients with sphincter dysfunction.[32]

**2) Hepatobiliary scintigraphy:** Dynamic hepatobiliary scintigraphy is a noninvasive method for the evaluation of SO functions. It provides indirect evidence of the increased sphincter resistance by measuring a significant delay in the hepatic uptake and washout.[33] It is highly sensitive as an early diagnostic tool for SOD,[34] provided a structural lesion of CBD has been excluded.[35] Sostre ***et al***.[33] have proposed a scoring system that combines the visual and quantitative criteria for the diagnosis of SOD using scintigraphy techniques after stimulation with cholecystokinin.

**3) Magnetic resonance cholangio-pancreaticography (MRCP):** Secretin MRCP is being studied as a noninvasive morphologic function test for the investigation of the dynamic anatomy of the pancreas.[36]

**4) Endoscopic retrograde cholangio-pancreaticography (ERCP):** Given the greater or comparable sensitivity of endoscopic ultrasonography (EUS) and MRCP for the exclusion of stones and other gross abnormalities, the National Institute of Health (NIH) State-of-the-Science Conference of January 2002 concluded that diagnostic ERCP alone, without the availability of SOM, is no longer advisable for investigating pain potentially of biliary or pancreatic origin.[37]

**5) SO manometry (SOM):** SOM is considered as the “gold standard” investigation for the evaluation of SO. Abnormally high basal sphincter pressure identified during SOM is regarded as the confirmation of the presumptive diagnosis of SOD.

*Significance of SOM:* A randomized, controlled study of patients with suspected type II biliary SOD predicts the improvement in pain after sphincterotomy. Patients with a basal pressure greater than 40 mm Hg had a clinical response rate of 91% compared with a 25% rate in patients with a high basal pressure, in whom a sham sphincterotomy was performed. For patients with a normal SO pressure, the response to sphincterotomy was only 42% and similar to that after sham sphincterotomy (33%).[38]

### Limitations of SOM

- Studies suggest that more easily measurable criteria such as elevated liver enzyme levels and biliary dilatation are superior in predicting a response to sphincter ablation.[39]
- Studies have also suggested that SOM may be highly sensitive for diagnosing SOD but may lack specificity.[38]

## APPROACH TO A CASE OF “PRESUMPTIVE” SOD

Most of the times, SOD is a diagnosis made by excluding other common causes of the aforementioned type of pain and hence the term “presumptive SOD.” Classic objective findings that suggest SOD include:[39]

- Dilated extra hepatic bile duct (usually >12 mm) or pancreatic duct (>6 mm in the head and 5 mm in the body) as per cross sectional imaging or cholangiography
- Transient biliary or pancreatic enzyme elevations (≥2 times the normal) during episodes of pain on at least two occasions, with a resolution over 24–48 h
- Delayed drainage of contrast medium from the bile duct (>45 min) or the pancreatic duct (>8 or 9 min) during ERCP

Two systems of classification, namely, the classical Milwaukee[40] and the modified Milwaukee, categorize “presumptive SOD” into three biliary and three pancreatic subtypes,.[39]

## TREATMENT

The objective of the treatment of SOD is to facilitate the drainage of pancreatic and biliary secretions into the duodenum. This can be achieved by:

1. Pharmacological treatment
2. Endoscopic approach
3. Surgery

**1. Pharmacological treatment:** Antispasmodics,[41] calcium channel blockers (Nifedipine) and nitrates have been used for SOD. Two short-term placebo-controlled, cross over studies showed that 75% of patients with suspected or documented SOD experienced statistically less pain with the use of oral nifedipine.[42,43] Octreotide, Prostaglandin E1, Gabexate and Botulinum toxin have also been suggested as useful relaxants of the hypertensive SOD.[44,45] In the light of safety of medical therapy and benign nature of SOD, medical therapy can be attempted in patients with type III and less severe type II SOD before a more invasive measure is contemplated.

**2. Endoscopic approach:** This includes sphincterotomy or stenting or both.

*Endoscopic sphincterotomy:* Most data on endoscopic sphincterotomy relate to biliary sphincter alone. Not much has been written regarding sphincterotomy for pancreatic type of SOD. In a postcholecystectomy patient presenting with presumed biliary type of SOD, the SO manometry (SOM) may be normal or abnormal. If SOM findings are abnormal, the relief of pain after sphincterotomy occurs in 90–95% of patients with type I SOD, 85% of those with type II SOD and 55–60% of those with type III disease.[46] When the SOM result is normal, pain relief after sphincterotomy still occurs in 90–95% of patients with type I SOD, 35–40% of patients with type II SOD and less than 10% of patients with type III SOD.[46] Thus, the following can be concluded.

- In biliary type I SOD, sphincterotomy can be empirically performed irrespective of SOM results
- SOM is necessary to predict the outcome in biliary type II SOD
- An abnormal SOM is mandatory before performing sphincterotomy in biliary type II SOD

Not many studies have addressed the problem of presumed SOD in patients with an intact gall bladder. An option in the evaluation of such patients is to assess the ejection fraction of the gall bladder and evaluate for fatty meal-stimulated bile duct dilatation. An abnormal gall bladder ejection fraction could be an indication for cholecystectomy, while a fatty meal-stimulated bile duct dilatation mandates SOM and possible sphincterotomy.[47] Of the patients who have documented SOD and an intact gall bladder and who are treated with sphincterotomy, first, only 43% have long-term pain relief; some additional patients eventually show response to cholecystectomy.[48]

The main limitation of an endoscopic approach for SOD is that it mainly addresses the biliary type of SOD. As a solution to this allegation, Park ***et al***.[49] have recommended an endoscopic dual pancreatobiliary sphincterotomy in patients with SOD associated with abnormal pancreatic basal pressure.

*Endoscopic stenting:* Stenting was used in patients with suspected SOD, but no increase in basal sphincter pressure, on the assumption that these patients may have intermittent spasms.[50] There was a poor symptomatic relief associated with high risk of stent-induced pancreatitis. Endoscopic stenting is no longer recommended as a routine method of treatment.

**3. Surgery:** The surgical procedure of transduodenal sphincteroplasty and transampullary septectomy has been standardized over the last two decades and is designed to provide adequate drainage of biliary and pancreatic ducts. The theoretical advantages of the surgical procedures over endoscopic approaches include the following:

- Access to the transampullary septum, which plays a major role in the pathogenesis of pancreatic type of SOD
- Accurate apposition to the duodenal and ductal mucosal lining, which avoids further scarring and stenosis of the ampulla

A recent series on transduodenal sphincteroplasty and transampullary septectomy claims good pain relief in 56%, moderate relief in 28% and poor outcome in 16% with a morbidity of 11%.[50]

## CONCLUSIONS

Sphincter of Oddi dysfunction is an uncommon condition; the definition and diagnosis of SOD are elusive and arbitrary and is a challenge from diagnostic and therapeutic point of view. The high failure rates of endoscopic and surgical treatments reflect the difficulties in accurate diagnosis and lack of specific objective criteria to select an appropriate therapy. Another reason for the high failure rates could be the fact that SOD could be a part of generalized smooth muscle disorder of the gastrointestinal tract. All the procedures on the sphincter should be undertaken with caution after meticulous investigations and patient selection should be based on strict objective criteria. Patients with suspected SOD are best managed by referral to centers with special expertise in the management of this condition.
